# Flavonoids and Asthma

**DOI:** 10.3390/nu5062128

**Published:** 2013-06-10

**Authors:** Toshio Tanaka, Ryo Takahashi

**Affiliations:** 1 Department of Clinical Application of Biologics, Osaka University Graduate School of Medicine, Osaka 565-0871, Japan; E-Mail: ryo723@athena.ocn.ne.jp; 2 Department of Immunopathology, WPI Immunology Frontier Research Center, Osaka University, Osaka 565-0871, Japan

**Keywords:** asthma, diet, flavonoids, prevention, treatment and management

## Abstract

Asthma is a chronic disease, characterized by airway inflammation, airflow limitation, hyper-reactivity and airway remodeling. It is believed that asthma is caused by the interaction between genetic and environmental factors. The prevalence of allergic diseases, including asthma, has increased worldwide during the past two decades. Although the precise reasons that have caused this increase remain unknown, dietary change is thought to be one of the environmental factors. Flavonoids, which are polyphenolic plant secondary metabolites ubiquitously present in vegetables, fruits and beverages, possess antioxidant and anti-allergic traits, as well as immune-modulating activities. Flavonoids are powerful antioxidants and anti-allergic nutrients that inhibit the release of chemical mediators, synthesis of Th2 type cytokines, such as interleukin (IL)-4 and IL-13, and CD40 ligand expression by high-affinity immunoglobulin E (IgE) receptor-expressing cells, such as mast cells and basophils. They also inhibit IL-4-induced signal transduction and affect the differentiation of naïve CD4+ T cells into effector T-cells through their inhibitory effect on the activation of the aryl hydrocarbon receptor. Various studies of flavonoids in asthmatic animal models have demonstrated their beneficial effects. The results of several epidemiological studies suggest that an increase in flavonoid intake is beneficial for asthma. Moreover, clinical trials of flavonoids have shown their ameliorative effects on symptoms related to asthma. However, these human studies are currently limited; further validation is required to clarify whether an appropriate intake of flavonoids may constitute dietary treatment and for part of a preventive strategy for asthma.

## 1. Introduction

Asthma is a chronic disease, characterized by airway inflammation, airflow limitation, hyper-reactivity and airway remodeling [1]. The development of inhaled corticosteroids and beta-adrenergic agonists have resulted in a paradigm shift for the treatment of asthma, while a greater understanding of the pathological mechanism of asthma is expected to lead to the development of other innovative drugs [2]. However, currently, asthma affects around 300 million individuals in the world [1], and the prevalence of asthma, as well as other allergic diseases, such as atopic dermatitis, allergic rhinitis and food allergy, has increased worldwide during the past two decades [3,4]. It is believed that the interaction between genetic and environmental factors causes individuals to become sensitized to environmental allergens and to suffer from allergic diseases [5,6,7,8]. Since foods and beverages contain both allergy-promoting and anti-allergic nutrients, it has been proposed that dietary change may be one of the environmental factors responsible for such an increase [9,10,11,12,13]. Vitamins A, C, D and E, minerals, such as selenium, copper, zinc and magnesium, probiotics and omega-3 polyunsaturated fatty acids (PUFAs), as well as polyphenols have been shown to possess anti-allergic properties, whereas omega-6 PUFAs are precursors for leukotriene C4, which is known to promote allergic inflammation. Flavonoids, on the other hand, which are polyphenolic plant secondary metabolites, can have powerful antioxidant, anti-allergic, anti-inflammatory and immune-modulating effects [14,15]. This review of recent findings discusses the possibility that an appropriate intake of flavonoids may play a role in the prevention and, eventually, in the management of asthma.

## 2. Biological Properties of Flavonoids

**Figure 1:**
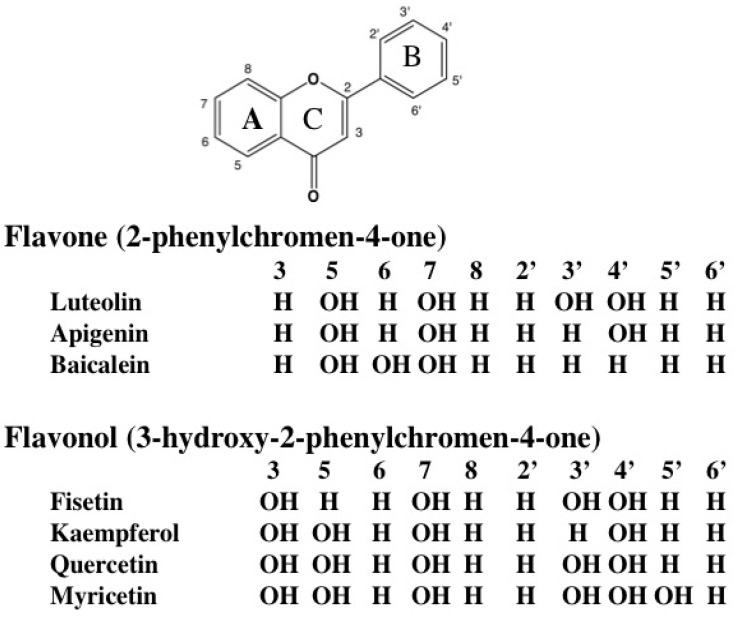
Structure of basic flavonoid skeletons and representative flavones and flavonols.

Flavonoids comprise a large group of low-molecular-weight polyphenolic plant metabolites that are found in fruits, vegetables, nuts, seeds, stems, flowers, roots, bark, dark chocolate, tea, wine and coffee and, thus, are common substances in the daily diet [14,15,16]. Flavonoids, which share a common structure consisting of two aromatic rings (A and B) that are bound together by three carbon atoms that form an oxygenated heterocycle (ring C), are classified into six subclasses: flavones (including luteolin, apigenin and baicalein), flavonols (fisetin, kaempferol, quercetin and myricetin) (Figure 1), flavanones (hesperetin, naringenin and eriodictyol), isoflavones (daidzein and genistein), anthocyanidins (cyanidin and pelargonidin) and flavanols (catechins and proanthocyanidins) [16]. Flavonoids have been found to have several biological effects, that is, antioxidant, anti-inflammatory, anticarcinogenic, anti-obesity, anti-diabetic and immune-modulating, and, also, to possess anti-allergic properties [14,15,16,17,18,19,20,21,22,23]. In addition, epidemiological evidence of the beneficial role of flavonoid intake in the fight against the risk of chronic diseases is promising [24,25].

There is strong evidence that an imbalance between the reducing and oxidizing systems in favor of a more oxidative state is present in asthma and that oxidative stress, associated with the presence of molecules, such as endogenous and exogenous reactive oxygen and nitrogen species, plays a significant role in airway inflammation and is one of the determinants of asthma severity [26,27,28]. Flavonoids are powerful antioxidants, since they stabilize the reactive oxygen species by reacting with the reactive compound of the radicals, scavenge nitric oxide and inhibit xanthine oxidase activity [29,30,31,32].

Immunoglobulin E (IgE)-mediated sensitization to domestic inhalant allergens, such as dust mites, cockroaches and pets is the most important risk factor for asthma, particularly in children [33]. IgE-mediated immune responses consist of a sensitization phase and an effector phase, both of which have been shown to be affected by the anti-allergic properties possessed by flavonoids. Fewtress and Gomperts first identified the inhibition by flavones of transport ATPase on histamine secretion from rat mast cells [34], which was followed by the discovery of the inhibitory effect of quercetin on allergen-stimulated human basophils [35,36]. Flavonoids were also found to inhibit hexosaminidase release from rat mast cells [37] and to suppress cysteinyl leukotriene synthesis through inhibition of phospholipase A_2_ (PLA2) and/or 5-lipoxygenase [38,39]. As for the suppressive effect of flavonoids on cytokine expression, luteolin, quercetin and baicalein were found to inhibit the secretion of granulocyte macrophage-colony stimulating factor (GM-CSF) by human cultured mast cells in response to cross-linkage of a high-affinity IgE receptor (FcεRI) [40], and it was subsequently demonstrated that these flavonoids also inhibit the IgE-mediated tumor necrosis factor (TNF)-α and interleukin (IL)-6 production by bone marrow-derived cultured murine mast cells [41]. In addition, we found that some flavonoids suppressed both IL-4 and IL-13 synthesis by allergen- or anti-IgE antibody-stimulated peripheral blood basophils [42,43,44]. Of the 45 known kinds of flavones, flavonols and their related compounds, luteolin, apigenin and fisetin, were the strongest inhibitors with the half-maximal inhibitory concentration (IC_50_) value of these flavonoids for inhibition of IL-4 synthesis ranging from 2.7 to 5.8 μM. Quercetin and kaempferol are representative of flavonoids associated with a substantial daily intake and were found to have a moderate inhibitory effect on IL-4 synthesis with an IC_50_ value of 15.7–18.8 μM, but myricetin showed no such effect, even at 30 μM. Luteolin, apigenin and fisetin inhibited IL-4 production by anti-CD3 antibody-stimulated T-cells, but at a relatively high dose (IC_50_ = 10–19 μM). Matsuda *et al*. [45] also reported that these three flavonoids inhibited IL-4 and TNF-α synthesis in a rat mast cell line, RBL-2H3. Similarly, luteolin, apigenin and fisetin were found to suppress CD40 ligand expression by activated basophils, whereas myricetin did not have such an effect [46]. In addition, we found that the inhibitory activity of flavonoids on IL-4 and CD40 ligand expression was mediated by their suppressive action on transcriptional factors, such as activator protein 1 (AP-1) and the nuclear factor of activated T-cells (NFAT) [42,47]. For the differentiation of B-cells into IgE producing cells, both the interaction of the CD40 ligand with CD40 and the effect of IL-4 or IL-13 on B cells are required [48], so that the inhibitory properties of flavonoids, such as luteolin, apigenin and fisetin, indicate that they are potentially natural IgE inhibitors. 

In addition to the inhibitory effect of flavonoids on IL-4 synthesis, kaempferol reportedly inhibits the activation of IL-4-induced signal transducer and activator of transcription (STAT)6 by specifically targeting Janus kinase (JAK)3 in hematopoietic cell lines, thus representing another anti-allergic activity of flavonoids [49].

The aryl hydrocarbon receptor (AhR) is a ligand-activated transcriptional factor that mediates the toxic and biological actions of many aromatic environmental pollutants, such as dioxins [50]. An AhR-based *in vitro* bioassay of the dioxin (2,3,7,8-tetrachlorodibenzo-*p*-dioxin [TCDD]) revealed that the flavonoids, apigenin, luteolin, baicalein, quercetin, kaempferol and myricetin, had noticeable inhibitory effects on AhR activation with an EC_70_ value (equal to 70% of the maximal response to TCDD) of 1.9–5.1 μM, while marked AhR activation was displayed, conversely, by daidzein, resveratrol, naringenin and baicalein, at higher concentrations [51]. It has recently been shown that AhR is a regulator of differentiation of naïve CD4+ T cells into effector T cell subsets [52,53,54,55], which suggests that flavonoids modulate immune functions through their binding to AhR.

Nuclear factor-kappaB (NF-κB) is one of the most important transcriptional factors that contribute pathologically to the development of asthma by inducing inflammatory and immune responses, cell adhesion and anti-apoptosis process [56]. Flavonoids are also known to inhibit NF-κB activation [57].

## 3. Epidemiological Studies on the Relationship between Flavonoid Intake and the Prevalence or Incidence of Asthma

Epidemiological studies have found that a high intake of fresh fruit and vegetables, both of which include flavonoids, may provide protection against asthma in adults and young adults [58,59]. Numerous cross-sectional or case-control studies also demonstrated the beneficial effect of fruit and vegetables on asthmatic symptoms or lung function in children in various countries and cities [60,61,62,63,64,65,66,67,68,69,70]. However, in the East Midlands and East of England regions of the UK, asthma prevalence was reportedly not associated with dietary fruit intake [71]. Higher maternal intake of fruit and vegetables was found to be associated with lower risk of children developing asthma [72], whereas higher maternal intake of green and yellow vegetables, citrus fruit and beta-carotene during pregnancy was significantly associated with a reduced risk of eczema, but not wheeze, in the offspring [73]. However, whether the beneficial effect of fruit and vegetables observed in these studies was due to flavonoids remained unknown. Moreover, a recent systematic review-based analysis provides evidence that the issues of confounding and effect modification have on the whole have been inadequately handled in observational epidemiologic studies investigating the role of diet in the development of asthma [74].

Few reports have dealt with direct associations between flavonoid intake and asthma. Shaheen *et al*. [75] reported that the results of a population-based case-control study of 607 cases and 864 controls in South London indicated that apple consumption and red wine intake were negatively associated with, respectively, asthma prevalence and severity, perhaps due to the protective effect of flavonoids, whereas a subsequent study [76] of dietary intake of catechins, flavonols and flavones did not find any significant associations with asthma prevalence or severity. A cohort epidemiological study of 10,054 adults in Finland concerning the association between flavonoid intake and risk of several chronic diseases found a reverse relationship between asthma incidence and higher quercetin, naringenin and hesperetin intakes [77]. Although there have been few reports of case-control or longitudinal studies examining direct associations between flavonoid intake and the prevalence or incidence of asthma, the findings of the epidemiological studies mentioned here suggest that higher flavonoid intake is beneficial for asthma. 

## 4. Efficacy of Flavonoids in Asthmatic Animal Models

Based on the anti-asthmatic characteristics of flavonoids observed *in vitro*, it was anticipated that administration of flavonoids might have beneficial effects on asthma, and indeed, various flavonoids have been shown to suppress airway inflammation and IgE response in asthmatic models.

As described elsewhere, luteolin, apigenin and fisetin are strong inhibitors of IL-4 synthesis. BALB/c mice were sensitized by intraperitoneal (i.p.) injection of ovalbumin (OVA) on days 0, 7 and 14, followed by daily aerosol inhalation of OVA beginning from days 19 to 23. Luteolin (0.1, 1.0 or 10 mg/kg) was administered orally and daily during the entire period, or after sensitization, 1 mg/kg was given orally from days 26 to 32. Both during and after sensitization, luteolin at the examined dose significantly suppressed OVA-induced airway bronchoconstriction and bronchial hyper-reactivity [78]. The administration of luteolin also resulted in a reduction in OVA-specific IgE levels in the sera and an increase in interferon gamma (IFN-γ) and a decrease of IL-4 and IL-5 levels in the bronchoalveolar lavage fluid (BALF). The preventative effect of luteolin and omega-3 PUFA supplement on airway responsiveness was also found in Ascaris suum-sensitized cats [79]. In an OVA-sensitized mouse model, apigenin, when administered i.p. at 5 or 10 mg/kg before the last OVA challenge, resulted in a significant inhibition of asthmatic reactions, such as serum IgE elevation, eosinophil accumulation, IL-4, IL-5 and eosinophil peroxidase (EPO) activity in BALF, as well as airway hyper-responsiveness [80]. Similar effects of apigenin at 2 or 20 mg/kg i.p. were observed in an OVA-sensitized model [81]. Intraperitoneal injection of fisetin at 3 mg/kg before OVA aerosol challenge was shown to attenuate lung inflammation, goblet cell hyperplasia and airway hyper-responsiveness [82]. The treatment also reduced expression of the key initiators of allergic airway inflammation (eotaxin-1 and thymic stromal lymphopoietin), Th2-associated cytokines (IL-4, IL-5 and IL-13) in lung tissues and Th2-predominant transcriptional factor GATA-3 and cytokines in thoracic lymph node cells and splenocytes. While it was previously reported that fisetin suppressed NF-κB activation [57,83], fisetin injection also impaired NF-κB activation in OVA-stimulated lung tissues. Moreover, when fisetin was injected intravenously at 0.3, 1 or 3 mg/kg before OVA aerosol challenge on days 22 to 24, it dose-dependently inhibited OVA-induced increases in total cell count, eosinophil count and IL-4, IL-5 and IL-13 levels in BALF [84]. It also attenuated OVA-induced lung tissue eosinophilia and airway mucus production, mRNA expression of adhesion molecules, chitinase, IL-17, IL-33, Muc5ac, a major airway glycoprotein and inducible nitric oxide synthase in lung tissues, as well as airway hyper-responsiveness. 

Quercetin and kaempferol are representative of flavonoids associated with a substantial daily intake and have a moderate inhibitory effect on IL-4 synthesis by activated basophils [42,43,44]. Oral administration of quercetin (10 mg/kg) or isoquercitrin (15 mg/kg) was found to suppress eosinophilic inflammation in lung homogenates in an OVA-immunized asthma model [85], while intraperitoneal injection of quercetin (8 or 16 mg/kg) reduced IL-4 expression and EPO activity, but increased IFN-γ expression in this model [86]. The effect of quercetin on asthmatic responses was also studied in OVA-sensitized conscious guinea pigs [87]. Quercetin (7.5 or 15 mg/kg, p.o.) significantly and dose-dependently inhibited both airway resistance on immediate-phase and late-phase response. Quercetin at the dose of 15 mg/kg also inhibited production of histamine, PLA2 and EPO. Single administration of kaempferol (10 or 20 mg/kg) could attenuate OVA challenge-elevated expression of eotaxin-1 and eosinophilic major basic protein via the blockade of NF-κB transactivation, thereby blunting eosinophil accumulation in airway and lung tissue [88]. Similarly, single intraperitoneal injection of sulfuretin (40 μg/kg) two hours after the last OVA challenge reduced airway inflammation, hyper-responsiveness and TNF-α, IL-5 and IL-13 expression in BALF, in association with the inhibition of the NF-κB signaling pathway [89]. Silibinin and sakuranetin also suppressed allergic airway inflammation via downregulation of NF-κB activity in an OVA-sensitized asthma model mouse [90,91].

It was also reported that nobiletin, a polymethoxyflavonoid, when administered i.p. to OVA-sensitized rats at a dose of 1.5 or 5 mg/kg before OVA aerosol challenge, reduced OVA-induced increases in eosinophils and eotaxin expression [92]. Subsequent investigations also found that flavonoids, such as 3-*O*-methylquercetin 5,7,3′,4′-*O*-tetraacetate [93], hesperidin [94], acacetin [95], chrysin [96], genistein [97] and skullcapflavone II [98], produced improvements in a mouse model of OVA-induced allergic asthma. Moreover, narirutin, when administered into OVA-sensitized NC/Nga mice orally at 10 mg/kg, significantly diminished OVA-induced airway inflammation and reduced eosinophilic counts in the peripheral blood and BALF, IL-4 level in BALF and serum IgE concentration [99]. The anti-asthmatic effect of limonene was examined in *Dermatophagoides farinae*-sensitized asthma models [100]. Intratracheal injection of limonene (1 mg/kg) during the entire experimental period inhibited airway hyper-responsiveness, airway remodeling and reduced the levels of IL-5, IL-13, eotaxin, monocyte chemotactic protein (MCP)-1 and transforming growth factor (TGF)-β in BALF. 

## 5. Human Intervention Studies of Flavonoids in Asthma

These findings regarding the *in vitro* and *in vivo* anti-allergic and anti-asthmatic properties of flavonoids strongly support the notion that an appropriate intake of flavonoids may constitute dietary treatment and/or a preventive strategy for asthma or other allergic diseases in humans [44,101,102,103]. Indeed, the results of recent clinical trials using flavonoid extracts or flavonoids indicate that flavonoids have beneficial effects on allergic rhinitis [104,105,106,107,108,109,110,111,112].

However, only a limited number of clinical trials of flavonoids for asthma have been performed. Pycnogenol, a proprietary mixture of water-soluble bioflavonoids, which is extracted from French maritime pine and contains proanthocyanidins, was found to be effective for asthma. The first trial was performed in a randomized, double-blinded, placebo-controlled, crossover design study of 26 patients with asthma of varying severity [113]. The patients were randomly assigned to receive either 1 mg/lb/day (maximum 200 mg/day) pycnogenol or placebo for the first period of four weeks and then to cross over to the alternate regimen for the next four weeks. Almost all of the 22 patients who completed the study responded favorably to pycnogenol, and the treatment led to a significant reduction in serum leukotrienes compared with response to the placebo. Subsequently, a randomized, placebo-controlled, double-blind study involving 60 subjects, aged 6–18 years, was performed over a period of three months to determine the effect of pycnogenol on mild-to-moderate asthma [114]. Compared with subjects taking the placebo, the pycnogenol group showed significantly greater improvement in pulmonary function and asthma symptoms in association with a significant reduction in urinary leukotrienes, which resulted in a reduction in or discontinuation of the use of rescue inhalers for the pycnogenol group. Another recent study, which assessed over a six-month period the efficacy of pycnogenol for improving allergic asthma management of patients with stable, controlled conditions, also showed favorable results [115]. In this study, a daily intake of 100 mg of pycnogenol proved to be effective for better control of signs and symptoms of allergic asthma and could reduce the need for medication. However, due to the small size and limited numbers of these trials and variability in outcomes, further clinical trials of pycnogenol are needed to establish its value for the treatment for asthma [116].

## 6. Conclusions and Perspectives

Asthma, a common disease worldwide, is the subject of growing concern, because of its increasing rate of prevalence [1]. It has been suggested that dietary changes may contribute to this increase [9,10,11,12,13]. Flavonoids possess anti-inflammatory, antioxidant, anti-allergic, as well as immune-modulating effects. Various studies of flavonoids in asthmatic models have shown their beneficial effects, whereas the evidence in epidemiological studies and human clinical trials is currently limited. Current findings regarding anti-asthmatic effects of flavonoids are summarized in Table 1. Recent development of databases of the flavonoid content of major vegetables, fruits and beverages, such as by the US Department of Agriculture (USDA) [117], the European BioActive Substances in Food Informative System (EuroFIR-BASIS) [118] and the Phenol-Explorer [119,120], can make a valuable contribution to epidemiological studies aimed at clarifying the relationship between flavonoid intake and the prevalence, incidence or severity of asthma. The Phenol-Explorer database was used to determine that the average total intake of flavonoids was 506 mg/day with 51 mg/day of flavonols and 33 mg/day of flavones in France [121], 370.2 mg/day with 24.8 mg/day of flavonols and 5.6 mg/day of flavones in Mediterranean countries and 373.7 mg/day with 29.5 mg/day of flavonols and 4.1 mg/day of flavones in non-Mediterranean countries [122]. Moreover, the EPIC (European Prospective Investigation into Cancer and Nutrition) study, which followed 477,123 subjects (29.8% men) aged 35–70 years old from 10 European countries to investigate the association between intake of flavonoids and lignans and incident gastric cancer, reported that the average intake of flavonoids for men and women was 445 mg/day and 434 mg/day with 26.5 mg/day and 26.7 mg/day of flavonols and 3.7 mg/day and 3.5 mg/day of flavones, respectively [123].

**Table 1:** Summary of anti-asthmatic effects of flavonoids.

*1. Biological properties* Antioxidant, anti-inflammatory, anti-allergic and immune-modulating activities.
*2. Hierarchy of inhibitory activity of representative flavonoids on IL-4 synthesis by basophils* Luteolin, apigenin, fisetin > kaempferol, quercetin > myricetin.
*3.* In vivo *effects in asthmatic animal models* Preventative and therapeutic beneficial effect of various flavonoids in several asthmatic models.
*4. Epidemiological study* An increase of flavonoid intake is suggested to be beneficial for asthma.
*5. Intervention study* Pycnogenol is efficacious for asthma.

Recent clinical trials have suggested that flavonoids can have a potent beneficial effect on allergic rhinitis, as well as asthma. Whether an appropriate intake of flavonoids can, in fact, constitute a dietary contribution to prevention and amelioration of asthma is, thus, an important issue for future studies. To this end, further well-designed, adequately powered trials are needed to determine the value of this dietary management component.
